# Supplementary material for: Modelling the mitigation of anti-vaccine opinion propagation to suppress epidemic spread: A computational approach
Source: PLoS One. 2025 Mar 20;20(3):e0318544. doi: 10.1371/journal.pone.0318544 (PMC11925286; doi:10.1371/journal.pone.0318544)
Supplement: S1 Appendix — (PDF) [file pone.0318544.s001.pdf]

Supplementary material to the paper: Modelling the mitigation of anti-vaccine opinion propagation to suppress epidemic spread: A computational approach

Sarah Alahmadi[1*], Rebecca Hoyle[2], Michael Head[3], Markus Brede[1],
[1] School of Electronics and Computer Science
[2] School of Mathematical Sciences
[3] Clinical Informatics Research Unit, Faculty of Medicine
University of Southampton, Southampton, United Kingdom
sha1a21@soton.ac.uk

Abstract

This document contains the supplementary materials for the paper "Modelling the mitigation of anti-vaccine opinion propagation to suppress epidemic spread: A computational approach".

The contents of this document are as follows:

- Comparison of Different Centrality Measures
- Disease Evolution

1 Comparison of Different Centrality Measures

In this section, we present the epidemic sizes obtained using different centrality metrics applied in our campaign to select the target set: betweenness centrality and degree centrality. As illustrated in Fig 1 below, the differences between the two metrics are minimal, with betweenness centrality showing slightly better performance.

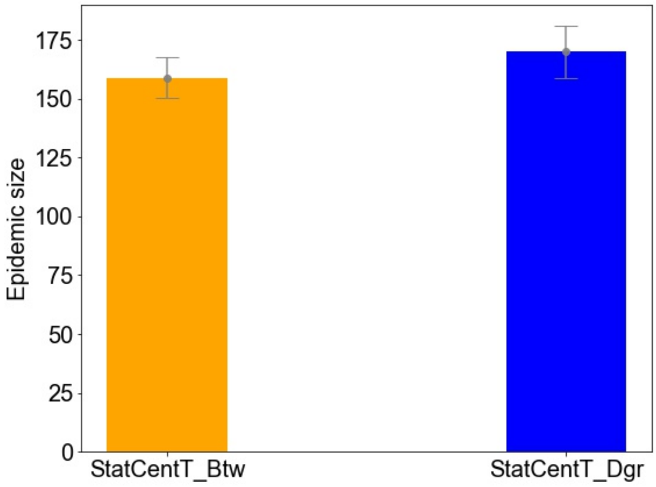

Figure 1: Comparison of epidemic size using different centrality measures: betweenness centrality (StatCentT_Btw) and degree centrality (StatCentT_Dgr). For all campaigns, $\mu^- = \mu^+ = 0.001$, the social rate is $\omega = 0.006$, the target set size is $T = 500$, and $\tau = \infty$. The results are the average of 100 simulations.

2 Disease Evolution

Figure 2 below illustrates the evolution of the disease after implementing the positive campaign in various ways. It shows the evolution of both the number of infected cases and the number of recovered cases. As shown, the peak of infected cases and the duration of the disease are significantly reduced when using dynamic campaigns, particularly DynRandT and DynAdvLocT, compared to the baseline StatRandAll, where the entire population is targeted randomly. These dynamic campaigns also outperform static campaigns, although the latter still perform better than StatRandAll.

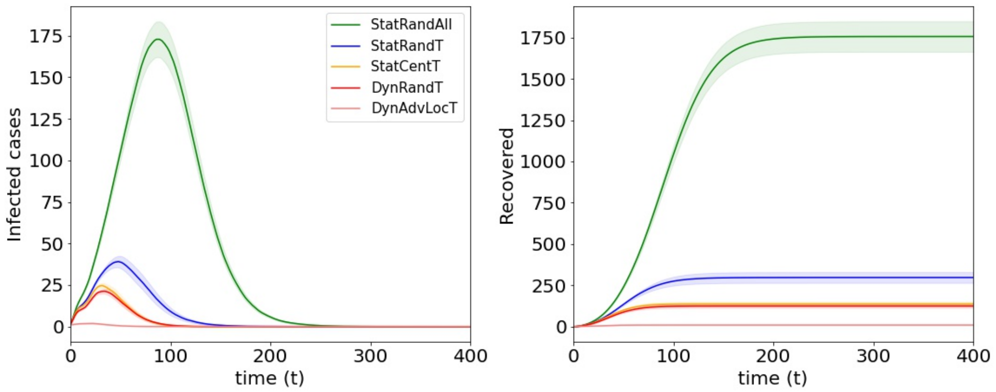

Figure 2: The disease evolution for the proposed campaigns with $\tau = \infty$. For all campaigns, $\mu^- = \mu^+ = 0.001$, the social rate is $\omega = 0.006$, and the target set size is $T = 500$ for static campaigns (i.e., StatRandT and StatCentT) and $T = 50$ for the other dynamic campaigns. For dynamic campaigns, the updating time is $t_r = 20$ for DynRandT, and $t_r = 1$ for DynAdvLocT campaign. Furthermore, for DynAdvLocT, $\zeta = Z = 10$. The results are the average of 50 simulations.
